# Chemerin 156F, generated by chymase cleavage of prochemerin, is elevated in joint fluids of arthritis patients

**DOI:** 10.1186/s13075-018-1615-y

**Published:** 2018-07-04

**Authors:** Lei Zhao, Yasuto Yamaguchi, Xiaomei Ge, William H. Robinson, John Morser, Lawrence L. K. Leung

**Affiliations:** 1 Department of Medicine, Division of Hematology, Stanford University School of Medicine, Stanford, CA 94305 USA; 2 Department of Medicine, Division of Endocrinology, Stanford University School of Medicine, Stanford, CA 94305 USA; 3 Veterans Affairs Palo Alto Health Care System, Room A4-131, Building 101, 3801 Miranda Avenue, Palo Alto, CA 94304 USA; 4 Department of Medicine, Division of Immunology and Rheumatology, Stanford University School of Medicine, Stanford, CA 94305 USA

**Keywords:** Adipokine, Arthritis, Inflammation, Osteoarthritis, Serine protease

## Abstract

**Background:**

Chemerin is a chemoattractant involved in immunity that also functions as an adipokine. Chemerin is secreted as an inactive precursor (chem163S), and its activation requires proteolytic cleavages at its C-terminus, involving proteases in coagulation, fibrinolysis, and inflammation. Previously, we found chem158K was the dominant chemerin form in synovial fluids from patients with arthritis. In this study, we aimed to characterize a distinct cleaved chemerin form, chem156F, in osteoarthritis (OA) and rheumatoid arthritis (RA).

**Methods:**

Purified chem156F was produced in transfected CHO cells. To quantify chem156F in OA and RA samples, we developed a specific ELISA for chem156F using antibody raised against a peptide representing the C-terminus of chem156F.

**Results:**

Ca^2+^ mobilization assays showed that the EC_50_ values for chem163S, chem156F, and chem157S were 252 ± 141 nM, 133 ± 41.5 nM, and 5.83 ± 2.48 nM, respectively. chem156F was more active than its precursor, chem163S, but very much less potent than chem157S, the most active chemerin form. Chymase was shown to be capable of cleaving chem163S at a relevant rate. Using the chem156F ELISA we found a substantial amount of chem156F present in synovial fluids from patients with OA and RA, 24.06 ± 5.51 ng/ml and 20.35 ± 5.19 ng/ml (mean ± SEM, *n* = 25) respectively, representing 20% of total chemerin in OA and 76.7% of chemerin in RA synovial fluids.

**Conclusions:**

Our data show that chymase cleavage of chem163S to partially active chem156F can be found in synovial fluids where it can play a role in modulation of the inflammation in joints.

**Electronic supplementary material:**

The online version of this article (10.1186/s13075-018-1615-y) contains supplementary material, which is available to authorized users.

## Background

Chemerin is involved in innate and adaptive immunity as a chemoattractant for natural killer (NK) cells, macrophages, and certain dendritic cell (DC) subsets [1, 2] but is also an adipokine [3–6]. Chemerin is well conserved across mammalian species with strong homology within its C-terminus. Human chemerin is expressed as a 163-amino-acid preproprotein containing a 20-amino-acid signal sequence, and is secreted as a 143-amino-acid precursor, prochemerin (chem163S) [2]. A characteristic of chemerin is that chem163S has low biological activity and requires precise extracellular C-terminal proteolytic processing to achieve its full functional activity. A variety of enzymes involved in blood coagulation, fibrinolysis, and inflammation—such as factor XIa, plasmin, carboxypeptidase B2 (also known as thrombin-activatable fibrinolysis inhibitor), elastase, or chymase—can proteolyze chemerin, leading to its activation or inactivation [7–10].

Rheumatoid arthritis (RA) and osteoarthritis (OA) are the most common forms of autoimmune and degenerative arthritis, respectively. Immune cell infiltrates are observed in synovial tissues in both diseases. We previously demonstrated that carboxypeptidase B2 (CPB2) regulates inflammation and tissue destruction in human and/or mouse models of RA and OA [11, 12]. In this study we investigate the presence of the cleaved isoforms of chemerin in RA and OA synovial fluid samples.

In order to study the biology of chemerin in vivo, we had previously produced various recombinant chemerin forms and developed specific ELISAs for them. Cleavage of the last four amino acids from chem163S by either FXIa or plasmin, giving rise to chem158K, represents a major step in prochemerin activation in vivo [13, 14]. Removal of the C-terminal lysine from chem158K, by either carboxypeptidase N (CPN) or CPB2, results in chem157S, the most potent chemerin form. On the other hand, chem155A is functionally inert [15]. A chemerin C-terminal peptide that represented chem156F is functionally active in vitro. In this article, we report the purification and characterization of chem156F, the generation of an anti-chem156F antibody, and the subsequent development of a specific ELISA for chem156F that we used to demonstrate the presence of chem156F in synovial fluid samples from patients with OA and RA.

## Methods

### Generation of CHO-S stable cell clones expressing human chem156F

A cDNA clone encoding human chemerin (NM_002889) was obtained from Open Biosystems (Huntsville, AL, USA). A cDNA fragment encoding chem156F was amplified by PCR using Platinum Pfx DNA polymerase (Invitrogen, Carlsbad, CA, USA) with termination codons inserted into the primers (CHEM-5 Ngo Primer 5′-AGCCGGCCACCATGCGACGGCTGCTGAT used as sense primer, and hCHEM-3 Nhe-F Primer 5′-AGCTAGCTTAGAAGGCGAACTGTCCAGGGA used as anti-sense primer) [13]. The cDNA encoding chem156F was cloned into a ubiquitous chromatin opening element (UCOE) vector, pCET-1019AS-puro, provided by Millipore. The plasmid encoding chem156F was transfected into CHO-S cells and subsequent selection was performed as described previously [15]. Plasmids encoding cDNAs for mouse chemerin forms, homologous to human chem156F (mouse mchem155F) and human chem157S (mouse chem156S), were produced in a similar fashion.

### Protein production

The selected CHO-S clone expressing chem156F was grown in suspension culture in CD Opti-CHO medium. To avoid cleavage of chemerin, the CHO-S cells were expanded in 225-cm^2^ T-flasks for 3 days before switching to CD-CHO medium. Static culture was then used to produce chem156F in 225-cm^2^ T-flasks for 3 days at 37 °C and purified as described previously [15]. The purified protein was characterized by SDS-PAGE. Mouse mchem156S and mchem155F were produced, purified, and characterized by the same methods.

### Calcium mobilization assays

L1.2 cells stably expressing human CMKLR1 were provided by Dr Brian Zabel and Dr Eugene C. Butcher (Stanford University School of Medicine and Veterans Affairs Palo Alto Health Care System) and cultured in RPMI 1640 with 10% FBS and 1 mg/ml geneticin [15]. Cells were labeled with Quest Fluo-8 AM dye, and then stimulated with recombinant chemerin forms in a 96-well plate calcium mobilization assay [15]. Each assay was repeated at least three times for each protein or peptide.

### Antibody preparation

Anti-human chemerin 156F (anti-chem156F) IgY was raised in chickens against the peptide sequence CZ^151^PGQFAF^156^ conjugated to KLH (Aves Labs, Tigard, OR, USA) and anti-chem156F IgY from eggs was purified by affinity chromatography to its cognate peptide bound to sepharose. To eliminate cross-reactivity with the chem157S form, negative selection affinity chromatography was performed by adsorption on an amino-linked (AminoLink kit; Thermo Scientific, Rockford, IL, USA) column coupled with the noncognate chemerin peptide KC^152^GQFAFS^157^. Rabbit polyclonal anti-human chemerin 163S (anti-chem163S), anti-human chemerin 158 K (anti-chem158K), anti-human chemerin 157S (anti-chem157S), and chicken polyclonal anti-human chemerin 155A (anti-chem155A) were prepared as described previously [14].

### Western blot analysis of the specificity of anti-human chem156F IgY

Purified recombinant chem163S, chem158K, chem157S, chem156F, and chem155A (200 ng each) were separated by SDS-PAGE under reducing conditions followed by western blot analysis with 500 ng/ml of the purified anti-chem156F IgY. The blot was developed with peroxidase-conjugated goat anti-chicken IgY antibody (100 ng/ml; Aves Labs) and detected using ECL (GE Healthcare, Sunnyvale, CA, USA).

### Specific ELISAs for chemerin forms

A rat monoclonal anti-human chemerin antibody (4 μg/ml; R&D Systems, Minneapolis, MN, USA) in PBS buffer was coated onto 96-well ELISA plates, and nonspecific binding sites were blocked with 1% BSA in PBS for 1 h before sample addition. Purified recombinant chem163S, chem158K, chem157S, chem156F, and chem155A were used as standards to construct calibration curves. Samples and standards were diluted with 1% BSA in PBS and incubated in the wells for 2 h. After washing with 0.05% Tween 20 in PBS, the samples were incubated with specific cognate antibodies (500 ng/ml) in PBS with 1% BSA for 1 h. In the case of anti-chem163S, chem158K peptide (10 μg/ml) was added to remove residual cross-reactivity with chem158K. In the case of anti-chem158K, chem163S peptide (10 μg/ml) was added to remove residual cross-reactivity with chem163S. In the case of anti-chem156F, chem155A peptide (10 μg/ml) was added to remove residual cross-reactivity with chem155A. For anti-chem155A, 10 μg/ml each of chem157S and chem158K peptides was added to remove the residual cross-reactivity with chem157S and chem158K. After washing with 0.05% Tween 20 in PBS, the samples were incubated with peroxidase-conjugated goat anti-rabbit IgG antibody (100 ng/ml) or peroxidase-conjugated goat anti-chicken IgY antibody (100 ng/ml) in PBS with 1% BSA for 1 h. After washing, tetramethylbenzidine substrate (Alpha Diagnostic International, San Antonio, TX, USA) was incubated for 10 min followed by the addition of Stop Solution (Alpha Diagnostic International) and absorbance at 450 nm measured. The concentrations of human chemerin forms were calculated from the calibration curves of the purified chemerin standards.

### Determination of chemerin forms in synovial fluid samples

Human synovial fluid samples were obtained under a protocol for discarded specimens approved by Stanford University School of Medicine or Partners Healthcare Institutional Review Boards. The samples were stored frozen at − 80 °C until the time of analysis. After thawing, 30 μl of a synovial fluid sample diluted to 400 μl with PBS was mixed with 50 μl of heparin-agarose (Sigma, St. Louis, MO, USA) and Complete Protease Inhibitor (Roche Applied Science, Pleasanton, CA, USA) [14], and the eluates were assayed by the specific ELISAs.

### Determination of chymase level in synovial fluid samples

Thawed synovial fluid samples were diluted 2-fold to 5-fold and assayed by human CMA1/Mast Cell Chymase ELISA kit (Life Span Biosciences Inc., Seattle, WA, USA).

### Kinetics of chymase cleavage of chem163S

Peptides (1–3 μM) representing the C-terminus of chem163S (chem163S-15mer) and chem156F (chem156F-8mer) were treated with chymase (30 nM) in PBS for 30 min at 37 °C before terminating the reaction with 100 nM PPACK. Then 100 μl of each reaction mixture was loaded onto a Zorbax Eclipse Plus C18 (4.6 mm × 150 mm) column (Agilent, Santa Clara, CA, USA) and separated with a 0–40% acetonitrile gradient in 0.1% trifluoroacetic acid (v/v) by HPLC. The concentration of peptides present in the reaction mixture was determined from the area under the curve for that peptide by comparison to a standard curve (1 μM–3 mM) of that peptide. The values for *K*_M_ and *k*_cat_ were determined by fitting to the Michaelis–Menten equation by nonlinear regression analysis using Prism v6 (Graphpad, San Diego, CA, USA). The experiments were performed three times independently, and the data were pooled for analysis.

### Statistics

Comparison of two samples was by Student’s *t* test; multigroup comparisons were by ANOVA followed by post hoc Kruskal–Wallis analysis. The analysis was carried out using Prism v7 (GraphPad, La Jolla, CA, USA). *p* < 0.05 was considered significant.

## Results

### Expression and purification of recombinant chem156F

DNA encoding chem156F was cloned into a plasmid, pCET-1019AS-puro, that has a UCOE element upstream of a guinea pig CMV promoter before transfection into CHO-S cells [15]. Two days after transfection, stable clones were selected by limiting dilution in 10 μg/ml puromycin, and 90 clones were screened for production of chem156F. A clone was identified that produced chem156F with a productivity of 4.74 pg/cell per day. Recombinant chem156F was purified by single-step cation exchange chromatography (Additional file 1: Figure S1A) and its C-terminus was confirmed by mass spectroscopy as described for purification and characterization of other recombinant chemerin forms [15]. The purified chem156F together with other purified recombinant human chemerins, chem163S, chem158K, chem157S, and chem155A, were shown to be > 95% pure by SDS-PAGE (Additional file 1: Figure S1B). Antibody raised against chem156F reacted only with chem156F (Additional file 1: Figure S1C).

### Functional characterization of chem156F

**Fig. 1:**
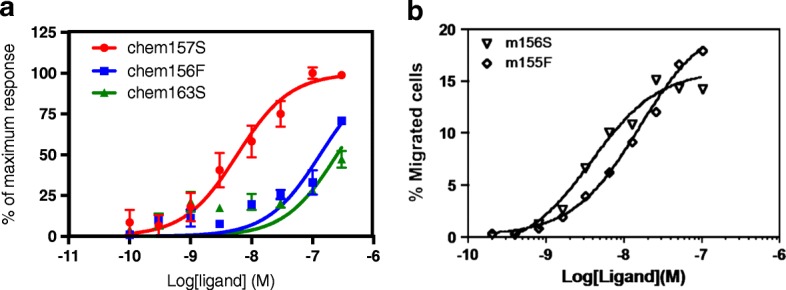
Biological activities of human chem156F and mouse mchem155F on CMKLR1. **a** Calcium flux in L1.2 cells transfected with human CMKLR1 in response to indicated concentration of chem156F (blue) and control recombinant proteins chem163S (green) and chem157S (red). **b** Indicated concentrations of mouse mchem156S (m156S, triangles) and mouse mchem156F (m155F, diamonds) assayed for their chemotactic activity on human CMKLR1/L1.2 cells using Transwell chemotaxis assay

We evaluated the potency of the purified chem156F in induction of calcium mobilization using CMKLR1-transfected L1.2 cells. Purified chem163S and chem157S were used as controls (Fig. 1a). The EC_50_ values for chem163S and chem156F were 252 ± 141 nM and 133 ± 41.5 nM respectively, while the EC_50_ value of chem157S was 5.83 ± 2.48 nM which is > 20-fold more potent than chem156F and > 40-fold more potent than chem163S. Thus, chem156F was about twice as active as the precursor, chem163S, but very much less active than chem157S, the most active form of chemerin based on the CMKLR1-transfected cell assay. The relative potencies of chem163S, chem156F, and chem157S were similar to that of their equivalent C-terminal peptides [15].

Unlike in mouse where mouse mchem156S, the homolog of human chem157S, and mouse mchem155F, the homolog of human chem156F, have approximately the same activity (data not shown), in humans chem156F is significantly less potent than chem157S. One explanation is that species differences between human and mouse CMKLR1 lead to chem156F being active on mouse but much less so on human CMKLR1. We tested this possibility by determining the potency of mouse mchem156S and mouse mchem155F in chemotaxis on L1.2 cells transfected with human CMKLR1. As on the L1.2 cells transfected with mouse CMKLR1, mouse mchem156S and mchem155F were equipotent showing that the difference in potency is not due to species-specific activity of chemerin on CMKLR1 cells (Fig. 1b).

### Generation and characterization of antibody specific for chem156F

In order to specifically detect chem156F in human samples, we prepared chicken anti-chemerin 156F (anti-chem156F) by immunization with a peptide representing the C-terminal sequence of chem156F. The specificity of the affinity-purified anti-chem156F IgY was demonstrated by western blot analysis in which anti-chem156F IgY only recognized its cognate protein but not the other four chemerin forms (Additional file 1: Figure S1C).

### Development of ELISAs specific for different human chemerin forms

**Fig. 2:**
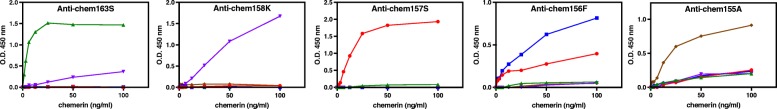
Characterization of specific antibodies against recombinant chemerin forms. Recombinant chem163S (green), chem158K (purple), chem157S (red), chem156F (blue), and chem155A (brown) by anti-chem163S, anti-chem158K, anti-chem157S, anti-chem156F, and anti-chem155A detected using specific ELISAs as described under “Methods”. O.D. optical density

Antibodies directed against the different forms of chemerin were used to develop sandwich ELISAs specific for the different chemerins, chem163S, chem158K, chem157S, chem156F, and chem155A. Recombinant chem163S, but not recombinant chem158K, chem157S, chem156F, or chem155A, was specifically detected in a dose-dependent manner by anti-chem163S, with a lower limit of detection of 0.5 ng/ml (Fig. 2). Likewise, anti-chem158K specifically recognized recombinant chem158K and not the other forms, whereas anti-chem157S and anti-chem155A specifically recognized recombinant chem157S and chem155A, respectively (Fig. 2). Anti-chem156F recognized recombinant chem156F, with some cross-reactivity against recombinant chem157S (Fig. 2). In order to measure chem156F accurately, taking into account the antibody’s cross-reactivity with chem157S, we used the signal from the chem156F ELISA to give the total concentration of [chem156F + chem157S] from which we then subtracted the chem157S concentration, as measured by the specific chem157S ELISA, to give the concentration of chem156F. Using this panel of ELISAs, the levels of different forms of human chemerin can be determined specifically.

### Detection of chemerin forms in the synovial fluid from arthritis

**Fig. 3:**
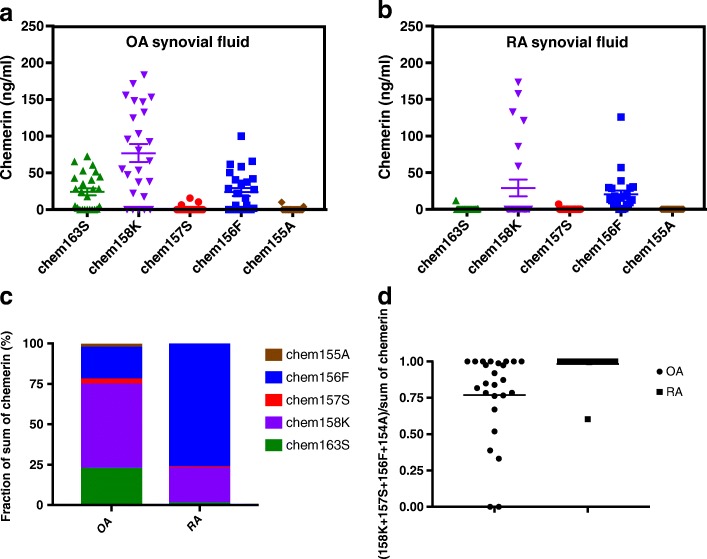
Levels of chemerin forms in synovial fluid of patients with RA and OA. **a, b** chem163S (), chem158K (), chem157S (), chem156F (), and chem155A () levels in synovial fluid of patients with **a** OA (*n* = 25) and **b** RA (*n* = 25) determined using specific ELISAs as described under “Methods”. Horizontal lines show mean ± SEM. **c** Chemerin forms in synovial fluid of OA and RA presented as percentage of sum of five specific ELISAs. **d** Ratio of cleaved chemerin levels (sum of chem158K, chem157S, chem156F, and chem155A) to sum of five chemerin forms in synovial fluid of OA and RA. OA osteoarthritis, RA rheumatoid arthritis

We have previously observed extensive proteolytic cleavage of chemerin (~ 75%) in synovial fluid samples from inflammatory arthritis patients and shown that chem158K is the dominant form [14]. To determine whether chem156F is present in the synovial fluid of arthritis patients, we investigated the levels of the five chemerin forms in synovial fluid samples from 25 patients with OA and 25 patients with RA. In OA synovial fluid samples, the levels of chem163S, chem158K, chem157S, chem156F, and chem155A were 24.28 ± 4.85 ng/ml, 77.04 ± 12.25 ng/ml, 1.31 ± 0.77 ng/ml, 24.06 ± 5.51 ng/ml, and 0.57 ± 0.43 ng/ml (mean ± SEM, *n* = 25) respectively (Fig. 3a). In RA synovial fluid samples, the corresponding levels were 3.06 ± 0.97 ng/ml, 40.57 ± 10.47 ng/ml, 0.29 ± 0.29 ng/ml, 20.35 ± 5.19 ng/ml, and 0 ± 0 ng/ml (mean ± SEM, *n* = 25) (Fig. 3b). In OA synovial fluid samples, when the fractions of individual chemerins from the sum of chemerin forms of each sample were averaged, 52% was chem158K, 20% was chem156F, while uncleaved chem163S was 23% (Fig. 3c). In RA joint fluid samples, the majority of chemerin was found to be chem156F (76.7%), with another 21.3% of chemerin presented as chem158K (Fig. 3c). The fraction of cleaved chemerins (chem158K + chem157S + chem156F + chem155A) was 75% in OA synovial fluid samples and 100% in RA synovial fluid samples (Fig. 3d), demonstrating that significant cleavage and processing of chemerin occurs in this extravascular compartment. Only minimal levels of chem157S and chem155A were detected in synovial fluids from either type of arthritis.

### Detection of chymase in synovial fluid from arthritis patients

**Fig. 4:**
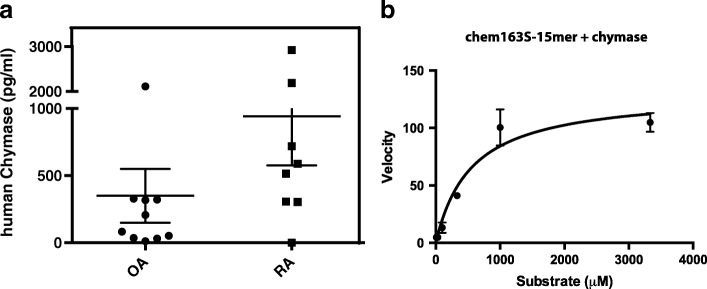
Chymase in synovial fluid of patients with RA and OA and its cleavage of chemerin. **a** Chymase levels in synovial fluid of patients with OA (*n* = 10) and RA (*n* = 8) determined using human chymase ELISA as described under “Methods”. **b** chem163S-15mer incubated with 10 nM chymase for 30 min before analysis by HPLC. Product concentration calculated by interpolation from standard curve, and velocities of product generation determined. OA osteoarthritis, RA rheumatoid arthritis

One enzyme that might generate chem156F from chem163S is chymase [8]. Therefore, we investigated chymase levels in OA and RA synovial fluid samples of arthritis patients. In OA and RA synovial fluid samples, the levels of chymase were 349.7 ± 200.2 pg/ml (*n* = 10) and 942.6 ± 366.8 pg/ml (*n* = 8) (mean ± SEM), respectively (Fig. 4a).

### Characterization of chem163S cleavage by chymase

To determine the kinetic constants for cleavage of chem163S by chymase, chem163S peptides consisting of the last 15 amino acids of the C-terminus of chemerin (chem163S-15mer) were subjected to chymase cleavage followed by HPLC analysis. Hydrolysis of chem163S-15mer by chymase showed *K*_M_ of 556.1 ± 174 μM, *k*_cat_ of 436.4 ± 44.96/min (mean ± SEM), and *k*_cat_/*K*_M_ of 0.78/μM/min (Fig. 4b and Table 1).

**Table 1:** Hydrolysis of chem163S peptides by chymase

Enzyme	Substrate	*K*_M_ (μM)	*k*_cat_ (/min)	*k*_cat_/*K*_M_ (/M/min)
Chymase	chem163S-15mer	556.1 ± 174	436.4 ± 44.96	7.8 × 10^5^

Substrates were digested with chymase as described in “Methods”. The values for *K*_M,_ *k*_cat_, and *k*_cat_/*K*_M_ were compared between peptides derived from chem163S. Data are mean ± SEM (*n* = 3).

## Discussion

In this study, we demonstrate that human chem156F is a distinct cleaved chemerin form. We show that chem156F is ~ 20-fold less potent than chem157S, the most potent form of active chemerin. Peptides representing the C-terminus of chem156F induced calcium mobilization and chemotaxis of CMKLR1/L1.2 cells to a similar degree to that observed with recombinant proteins [15], confirming that chem156F is not as potent as chem157S. This finding is different from that observed in the mouse where the corresponding mouse homologs mchem156S and mchem155F are equipotent (Zhao et al., PLoS One, submitted). The fact that these two mouse chemerins are also equipotent using the human CMKLR1-transfected cells indicates that the difference in potency is most likely not at the level of CMKLR1, the chemerin receptor. Instead, it implies that the difference resides in the proteins and peptides. As the chem156F peptide has similar potency to the chem156F protein, this shows that it must be a sequence difference within the C-terminal tail that leads to the differences in activity. The c-terminal sequence for human chem156F is AGEDPH**SFYF**PGQFAF and for mouse mchem155F is AGEDPH**GYFL**PGQFAF (amino acids in bold representing substitutions). Thus, mouse mchem155F may present the C-terminus in a better conformation for binding to CMKLR1 than does human chem156F.

In both OA and RA joint fluid samples, there is evidence of extensive activation and cleavage of prochemerin, with chem158K being the dominant form and minimal levels of chem157S [14]. Here we extend that observation by showing that there is a substantial amount of chem156F present in both OA and RA samples, with a strong trend toward increased chem156F in RA as compared to OA synovial fluids. The differential expression levels observed correlate with the lower degree of synovial inflammation in OA as compared to RA [16]. The expression, cleavage, and activation of chemerin in synovial tissues could contribute to the trafficking and activation of immune cells in RA and OA. Circulating chemerin levels in RA have been associated with endothelial cell activation and atherosclerosis [17].

There is immunohistological evidence for expression of prochemerin on endothelial cells and synovial lining in the synovium and its expression on fibroblast-like synoviocytes is upregulated by TNF-α and IFN-γ [18]. Human synovial fibroblasts from patients with RA and OA express both chemerin and its receptor CMKLR1, and chemerin upregulates CCL2 in synovial fibroblasts [15]. Human chondrocytes express both chemerin and CMKLR1, and when treated with chemerin respond by producing inflammatory cytokines such as IL-1β [19], while chondrocytes increase chemerin production upon IL-1β treatment [20]. This suggests that chemerin may play a role in cartilage damage by increasing local inflammation.

A relationship has been found between disease severity and chemerin levels in OA [21, 22] and in patients with temperomandibular disorders [23], supporting a role for chemerin in OA. In RA, synovial fluid contains chemerin [24]. In none of these studies was the chemerin characterized in terms of the presence of different forms.

Based on the differences between plasma chemerin species and those found in the synovium [13, 14], enzymatic cleavage of prochemerin is probably occurring locally within the joint space. Mast cell chymase is detectable within the same joint fluid samples in which chem156F was found and it cleaves prochemerin chem163S into chem156F with kinetic constants similar to those of other substrates [25, 26]. Mast cells accumulate in arthritic joint tissues of RA patients [27], and mice deficient in either mast cell protease-4 (mMCP-4) or mMCP-5, the two mouse chymases that are the closest functional and structural homologs to the single human chymase, display significantly reduced inflammation in immune arthritis models [28, 29].

Chemerin is also an adipokine and activated chemerin can be found in adipose tissues [30, 31]. Mast cells are also present in adipose tissues and have been shown to release chymase [32, 33], suggesting that mast-cell-derived chymase may be contributing to the activation of chemerin in adipose tissue.

The other dominant form of chemerin in synovial fluids of arthritis, chem158K, can be generated by FXIa, plasmin, or tryptase cleavage of chem163S and is modestly active [14, 15, 30]. The chem158K levels present in the synovial fluid samples may be sufficient to contribute a significant amount of chemerin activity without further processing, or may be further cleaved to chem157S or chem156F. We have previously shown that fibroblast-like synoviocytes express procarboxypeptidase B2 (proCPB2) and there is functional thrombomodulin on these cells that supports thrombin activation of proCPB2 to active CPB2 [34]. CPB2 should convert chem158K into chem157S [13], but minimal chem157S was detected in the RA and OA joint fluid samples (Fig. 3), similar to our previous observation [14]. It is likely that chem157S is further proteolyzed, possibly to chem155A but since chem155A is present at a very low level this may be unlikely, but also to other degraded forms, such as those found in the plasma of patients undergoing bariatric surgery [30].

**Fig. 5:**
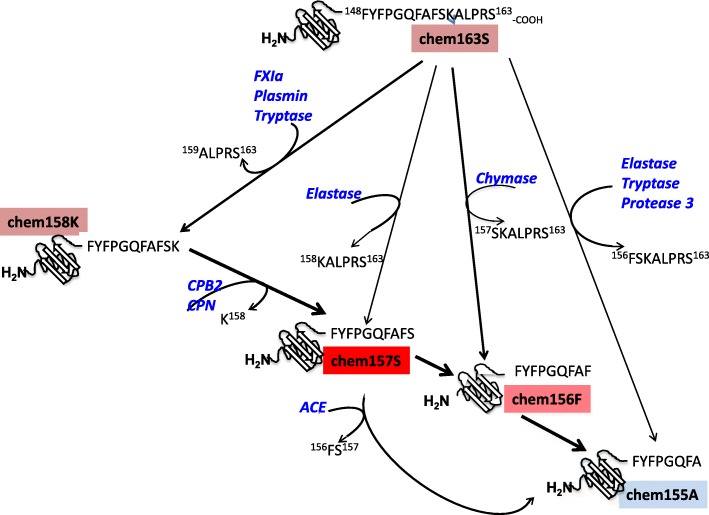
Schematic of chemerin cleavages. Chem163S (pink box) can be cleaved either by factor XIa (FXIa), Plasmin, or Tryptase generating chem158K (pink box), by elastase generating a mixture of chem157S (red box) and chem155A (blue box), by chymase generating chem156F (dark pink box), or by proteinase 3 and Tryptase generating chem155A. chem158K can then be cleaved by carboxypeptidase B2 (CPB2) or carboxypeptidase N (CPN) to form active chem157S, which is subsequently inactivated by angiotensin-converting enzyme (ACE) forming inactive chem155A

Prochemerin (chem163S) can be cleaved in its C-terminal domain by different serine and cysteine proteases in vitro, generating a series of chemerin forms with different levels of activity (Fig. 5). The pathway(s) by which chemerin is activated and inactivated may depend on the location with plasma being different from tissues such as synovial fluid and adipose tissue [13, 30].

## Conclusions

In this study, we produced and purified chem156F, and demonstrated its presence in synovial fluids of arthritis patients. This chemerin form and the specific antibody generated will enable further characterization of the proteolytic cleavages of prochemerin. Taken together with other studies of chemerin, these data suggest that in vivo in both humans and mice different proteolytic cleavage pathways occur in different tissue compartments to regulate prochemerin activation and inactivation [13, 30]. Our findings identify a new cleaved form of chemerin, chem156F, that could promote inflammation and tissue destruction in RA and OA. Future studies are needed to further define the potential mechanistic role of chem156F and other isoforms of chemerin in RA and OA.

## Additional file

Additional file 1: **Figure S1.** Purification and characterization of chem156F. Following centrifugation and filtration, conditioned cell culture medium applied to an anion exchange column equilibrated with PBS (pH 7.4) and then chem156F protein eluted by a gradient of increasing ionic strength. **A** Absorbance at 280 nm (mAU, blue line), NaCl gradient from 150 to 500 mM (green), and conductivity (brown) of purification of hchem156F. **B** Coomassie Blue stained SDS-PAGE of purified recombinant chem163S (lane 1), chem158K (lane 2), chem157S (lane 3), chem156F (lane 4), and chem155A (lane 5). **C** Anti-chem156F specific for chem156F characterized by western blot analysis of recombinant chem163S (lane 1), chem158K (lane 2), chem157S (lane 3), chem156F (lane 4), and chem155A (lane 5), as described under “Methods”. Molecular mass markers shown on left. (PDF 131 kb)

## Abbreviations

CMKLR1: Chemokine-like receptor 1
CPB2: Carboxypeptidase B2
CPN: Carboxypeptidase N
mMCP: Mouse mast cell protease
OA: Osteoarthritis
proCPB2: Procarboxypeptidase B2
RA: Rheumatoid arthritis
UCOE: Universal chromosome opening element
