# Supplementary material for: Chemerin 156F, generated by chymase cleavage of prochemerin, is elevated in joint fluids of arthritis patients
Source: Arthritis Res Ther. 2018 Jul 4;20:132. doi: 10.1186/s13075-018-1615-y (PMC6033211; doi:10.1186/s13075-018-1615-y)

Additional file 1. Purification and characterization of chem156F.

Following centrifugation and filtration, conditioned cell culture medium was applied to an anion exchange column equilibrated with PBS (pH 7.4) and then chem156F protein was eluted by a gradient of increasing ionic strength. **A**, absorbance at 280 nm (mAU, *blue line*), NaCl gradient from 150 to 500 mM (*green),* and conductivity (*brown*) of the purification of hchem156F. **B**, coomassie blue stained SDS-PAGE of purified recombinant chem163S (lane 1), chem158K (lane 2), chem157S (lane 3), chem156F (lane 4), and chem155A (lane 5). **C,** anti-chem156F specific for chem156F was characterized by Western blot analysis of recombinant hchem163S (lane 1), chem158K (lane 2), chem157S (lane 3), chem156F (lane 4), and chem155A (lane 5), as described under "Experimental Procedures". Molecular mass markers are shown on the left of the panels.

A.

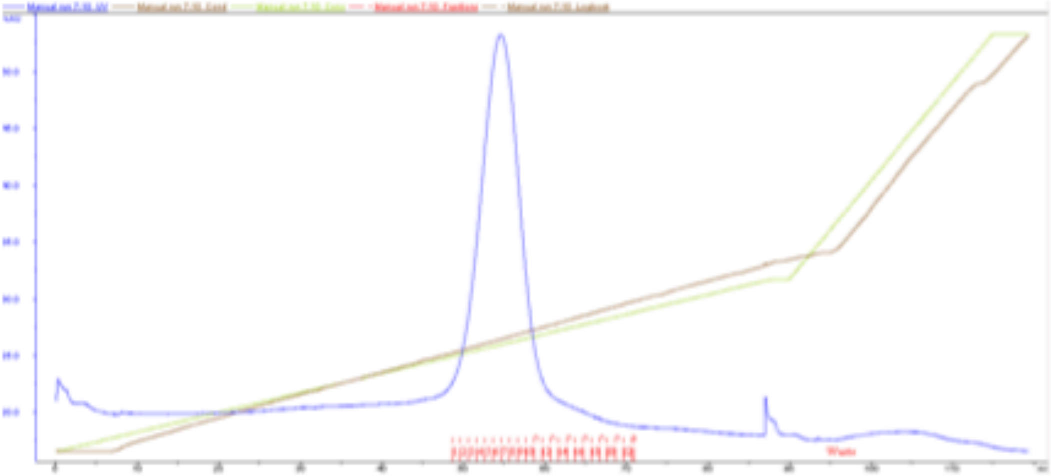

B.

C.

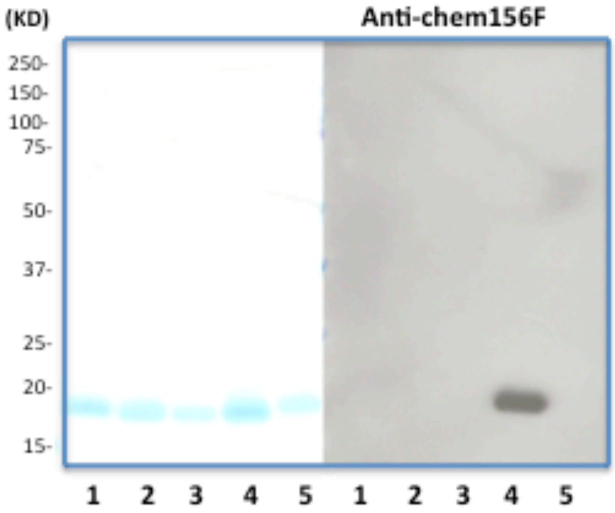

Supplement: Supplementary file 1 — Figure S1. Purification and characterization of chem156F. Following centrifugation and filtration, conditioned cell culture medium applied to an anion exchange column equilibrated with PBS (pH 7.4) and then chem156F protein eluted by a gradient of increasing ionic strength. A Absorbance at 280 nm (mAU, blue line), NaCl gradient from 150 to 500 mM (green), and conductivity (brown) of purification of hchem156F. B Coomassie Blue stained SDS-PAGE of purified recombinant chem163S (lane 1), chem158K (lane 2), chem157S (lane 3), chem156F (lane 4), and chem155A (lane 5). C Anti-chem156F specific for chem156F characterized by western blot analysis of recombinant chem163S (lane 1), chem158K (lane 2), chem157S (lane 3), chem156F (lane 4), and chem155A (lane 5), as described under “Methods”. Molecular mass markers shown on left. (PDF 131 kb) [file 13075_2018_1615_MOESM1_ESM.pdf]
